# Capturing the whole-school food environment in primary schools

**DOI:** 10.1017/S1368980023001131

**Published:** 2023-08

**Authors:** Sarah E Moore, Sarah F Brennan, Fiona Lavelle, Moira Dean, Michelle C McKinley, Dilara Olgacher, Patrick McCole, Ruth F Hunter, Laura Dunne, Niamh E O’Connell, Chris T Elliott, Danielle McCarthy, Jayne V Woodside

**Affiliations:** 1 Institute for Global Food Security, Queen’s University Belfast, Belfast BT9 5AG, United Kingdom of Great Britain and Northern Ireland; 2 Centre for Public Health, Queen’s University Belfast, Belfast BT12 6BA, United Kingdom of Great Britain and Northern Ireland; 3 Queen’s Management School, Queen’s University Belfast, Belfast BT9 5EE, United Kingdom of Great Britain and Northern Ireland; 4 Centre for Evidence and Social Innovation, Queen’s University Belfast, Belfast, United Kingdom of Great Britain and Northern Ireland

**Keywords:** School, Food environment, Whole-school approach, Canteen, Children, Diet, Nutrition

## Abstract

**Objective:**

The school food environment (SFE) is an ideal setting for encouraging healthy dietary behaviour. We aimed to develop an instrument to assess whole-SFE, test the instrument in the school setting and demonstrate its use to make food environment recommendations.

**Design:**

SFE literature and UK school food guidance were searched to inform instrument items. The instrument consisted of (i) an observation proforma capturing canteen areas systems, food presentation and monitoring of food intake and (ii) a questionnaire assessing food policies, provision and activities. The instrument was tested in schools and used to develop SFE recommendations. Descriptive analyses enabled narrative discussion.

**Setting:**

Primary schools.

**Participants:**

An observation was undertaken at schools in urban and rural geographical regions of Northern Ireland of varying socio-economic status (*n* 18). School senior management completed the questionnaire with input from school caterers (*n* 16).

**Results:**

The instrument captured desired detail and potential instrument modifications were identified. SFE varied. Differences existed between food policies and how policies were implemented and monitored. At many schools, there was scope to enhance physical eating environments (*n* 12, 67 %) and food presentation (*n* 15, 83 %); emphasise healthy eating through food activities (*n* 7, 78 %) and increase parental engagement in school food (*n* 9, 56 %).

**Conclusions:**

The developed instrument can measure whole-SFE in primary schools and also enabled identification of recommendations to enhance SFE. Further assessment and adaptation of the instrument are required to enable future use as a research tool or for self-assessment use by schools.

The school food environment (SFE) is a potential setting for encouraging children’s healthy dietary behaviour^(1)^. SFE are complex, influenced by many interconnected elements, including infrastructure, school staff, parents and wider community stakeholders and organisations^(2)^. Some definitions of the term SFE focus on food available through the canteen, vending machines and tuckshops^(3,4)^; other definitions are broader, which, in addition to food available, also encompass the physical space, infrastructure and conditions where food is available^(5)^. A ‘whole-school’ food approach to encouraging healthy dietary behaviour is advocated internationally. As well as education, this includes all school food provision, policies on food provided and brought from home, the physical school setting and parental engagement^(6,7)^. Existing instruments to assess SFE do not use this holistic definition, commonly assessing specific settings and aspects of the SFE, most often the canteen and physical attributes (e.g. presence of dining facilities and food provided)^(8)^. Therefore, the extent to which schools adopt a ‘whole-school’ food environment (WSFE) is unclear. This research aimed to (i) develop a research instrument to capture WSFE; (ii) test the instrument in the primary school setting and (iii) demonstrate how the instrument can be used to identify areas of good practice or to be enhanced when implementing WSFE.

## Methodology

A search of SFE literature and guidance (non-systematic) informed included instrument items. The search was conducted by one member of the research team in July 2018 using online databases (PubMed, Medline and Web of Science) and a search engine (Google). Broad search terms such as ‘school food environment’ and ‘assessing the school food environment’ were used to capture scientific publications of SFE interventions or experimental studies and measures of the SFE. UK Government web pages were visited directly to obtain school food standard policy documents and guidance documents for implementing the standards. Based on this search, an observation proforma and questionnaire were developed (see online supplementary material). The 16-item observation proforma captured detail on canteen areas, atmosphere, systems, food presentation and monitoring of pupil food intake, based on guidance for improving the school dining experience^(9)^ and was completed by researchers. A 27-item questionnaire assessed school food policies, provision and activities, adapted from a previous school environment questionnaire^(10)^ and was completed by school senior management/catering staff. Four research team members with experience of school-based nutrition research independently reviewed instrument items to assess face validity (i.e. that instrument items appeared to assess what was intended, were not assessing overlapping concepts and covered all aspects of the WSFE)^(11)^. Face validity was established as it was agreed that these criteria were met^(11)^ and therefore the instrument design was deemed appropriate. The instrument has not yet undergone further validity and reliability assessment as this stage of the research aimed to conduct an initial pilot test of the instrument for collecting WSFE data.

The instrument was pilot-tested to collect WSFE data as a secondary outcome of a food-based randomised-controlled trial in primary schools (Project DAIRE)^(12)^. Food environment data were collected October 2018 to March 2019. A researcher at each school canteen completed the observation proforma during lunch and the questionnaire was administered. Data were entered into SPSS (version 26) and descriptive analyses conducted. Areas of strength in implementing a WSFE (implemented by > 50 % of schools) and areas that could be enhanced (implemented by ≤ 50 %) were identified. Recommendations for optimising WSFE were developed based on these and school food guidance^(9,13)^.

## Results

### Testing the instrument to capture whole-school food environment

An observation was undertaken at all DAIRE schools (*n* 18). The questionnaire was completed by *n* 16 schools. Participating schools ranged in size (34–740 pupils) and geographical areas (urban *n* 13; rural *n* 5).

### Physical setting

School canteens were multi-purpose halls or classrooms (*n* 10, 56 %) or separate facilities within school buildings/grounds (*n* 8, 44 %). At most schools, (*n* 15, 83 %) pupils eating school meals and packed lunches ate at separate tables or, at a few schools (*n* 2, 11 %), in separate rooms (Table 1). Eating areas were clean (*n* 18, 100 %) and largely spacious (*n* 15, 83 %). At some schools (*n* 6, 33 %), eating areas were deliberately welcoming, e.g. with artwork, bunting and table centrepieces. Most eating area atmospheres (*n* 14, 78 %) were busy and noisy (children chatting). Lunch periods were on average 27·5 min and a sense of rush for pupils to finish lunch was sometimes apparent (*n* 7, 39 %).

**Table 1:** Observations of the food environments of primary schools in Northern Ireland (*n* 18)

		Schools	
School food environment observations		*n*	%
Canteen area	Welcoming eating area?		
	Yes	6	33·3
	Scope to enhance	12	66·7
	Clean eating area?		
	Yes	18	100·0
	Scope to enhance	0	
	Adequate space and seating in eating area?		
	Yes	15	83·3
	Scope to enhance	3	16·7
	Busy atmosphere in eating area?		
	Yes	14	77·8
	No	4	22·2
	Ambient temperature in eating area?		
	Yes	11	61·1
	Scope to enhance	7	38·9
	Ambient lighting in eating area?		
	Yes	15	83·3
	Scope to enhance	3	16·7
	Pleasant smell in eating area?		
	Yes	5	27·8
	No distinct smell	13	72·2
Canteen systems	Do pupils queue?		
	Yes, all pupils	17	94·4
	Younger pupils are served, older pupils queue	1	5·6
	Reasonable queue time?		
	Yes	17	94·4
	Scope for improvement	1	5·6
	Multiple serving points?		
	Yes	8	44·4
	No	10	55·6
	Arranged seating?		
	Yes, packed lunch and school meal pupils seated separately	15	83·3
	No	1	5·6
	Packed lunch pupils eat in separate room to dining area	2	11·1
	Sense of rush to eat?		
	Yes	7	38·9
	No	11	61·1
Display and presentation of food	Daily menu displayed?		
	Yes	7	38·9
	No	11	61·1
	Menu for term displayed?		
	Yes	15	83·3
	No	3	16·7
	Foods labelled?		
	Yes	0	
	No	18	100·0
	Foods displayed?		
	Yes	3	16·7
	No	15	83·3
	What is food served on?		
	Segmented tray	17	94·4
	Plate	1	5·6
	Appealing food presentation?		
	Yes	3	16·7
	Scope to enhance	15	83·3
	Choice of meal available?		
	Yes	16	88·9
	No, set meal	2	11·1
Encouraging eating and promotion of nutrition messages	Teaching staff eating school meals in canteen?		
	Yes	4	22·2
	No	14	77·8
	Monitoring/ encouraging pupil food intake?		
	Yes	8	44·4
	No/ not clear	9	50·0)/ 1 (5·6)
	Healthy eating posters displayed?		
	Yes	13	72·7
	No	5	27·8

School meal queue systems were well controlled by teaching/canteen staff, and queue times were perceived as ‘reasonable’ (*n* 17, 94 %). Pupils had a choice of two meals at most schools (*n* 16, 89 %). Many schools displayed the term’s meal menu (*n* 15, 83 %); some also displayed the daily menu (*n* 7, 39 %). Foods on offer were not labelled (*n* 18, 100 %). Some schools (*n* 3, 17 %) displayed foods using serveware and creative presentation, such as cutting up fruit.

### Food policies

Most schools (*n* 14, 88 %) already had a healthy eating policy or were developing one (*n* 2, 13 %), and policies were communicated to parents (*n* 13, 81 %) (Table 2). Some break-time snack policies were communicated as guidance (*n* 7, 44 %), whereas others were requirements (*n* 6, 38 %). Policies on suitable packed lunch foods were usually communicated as guidance (*n* 10, 63 %), rather than requirements. Pupil adherence to policy guidance/requirements was encouraged at some schools (*n* 7, 44 %), whereas other schools (*n* 4, 25 %) enforced adherence through contacting parents or not allowing pupils to eat non-adherent foods brought into school.

**Table 2:** School food environment questionnaire completed by principals and catering staff in primary schools in NI (*n* 16)

Healthy eating policies	*n*	%	Food activities	*n*	%	Food activities	*n*	%	School meals	*n*	%
Existing healthy eating policy?			Have existing breakfast club?	14	87·5	Themed food days?			Average % taking school meals/ day?		
Yes, school specific	13	81·3	Types of food served at breakfast?			Yes, regularly	3	18·8	41–50	3	18·8
Yes, general	1	6·3	Cereal and toast/bread**s**	14	100·0	Yes, *ad hoc*	11	68·8	51–60	4	25·0
In development	2	12·5	Fruit**/**veg (including juice)	8	57·1	No	2	12·5	61–70	3	18·8
			Milk/ cheese/ yoghurt	6	42·9				71–80	3	18·8
			Eggs/ sausages	3	21·4				81–90	1	6·3
			Water	1	7·1				Not stated	2	12·5
			Other drink (hot drink/ juice unspecified)	10	71·4						
Years healthy eating policy in place?			% total n pupils attending breakfast club?			Types of themed food days?			% of FSM entitled pupils taking meals?		
In development	2	12·5	5–10	5	35·7	Special event	5	35·7	0–25	1	6·3
0–10 years	11	68·8	11–20	5	35·7	Food from other cultures	3	21·4	26–50	2	12·5
11–20 years	3	18·8	21–25	3	21·4	Linked with curricular topics	1	7·1	51–75	3	18·8
			Not stated	1	7·1	Combination of the above	4	28·6	76–100	8	50·0
						Not stated	1	7·1	Not stated	2	12·5
Policy communicated to parents?			Food provision at break?			Chef/ cookery demonstrations?	5	31·3	Does menu choice impact n taking meals?		
Yes	13	81·3	Yes, all pupils	8	50·0	Yes, healthy meals *ad hoc*	1	20·0	Yes	15	93·4
In development	2	12·5	Yes, nursery and younger pupils	3	18·8	Yes, healthy snacks *ad hoc*	3	60·0	Not stated	1	6·3
Not stated	1	6·3	No	5	31·3	Yes, produce from school allotment *ad hoc*	1	20·0			
Method policy communicated?			Types of food provided at break?			Cookery club?			Foods stated as popular menu items		
Verbal communication	11	84·6	Fruit	5	45·5	Yes, regularly	5	31·3	Chips	12	75·0
School newsletter	10	76·9	Fruit and healthy drinks, e.g. milk/ water	1	9·1	Yes, *ad hoc*	4	25·0	Chicken curry	6	37·5
Paper or electronic copy	9	69·2	Fruit and bread/toast	3	27·3	No	7	43·8	Roast dinner	6	37·5
Posters/signs around school	9	69·2	Fruit and yoghurt	1	9·1	What do pupils make at cookery club?			Pizza	5	31·3
School website/ social media	7	53·8	Fruit and crackers and cheese	1	9·1	Healthy meals/ snacks	2	22·2	Burger	5	31·3
Texts to remind parents	1	7·7				Less healthy meals/ snacks	2	22·2	Sausages	3	18·8
Not stated	1	7·7				Healthy and less healthy meals/snacks	5	55·6	Chicken goujons/ nuggets	3	18·8
Does policy include packed lunch advice?			Other food provision			Does your school use a garden/ allotment?			% food waste estimated in canteen?		
Yes guidance	10	62·5	Afterschool (fruit)	1	6·3	Education	2	12·5	0–10	5	31·3
Yes requirements	3	18·8	Supper club	0	0·0	Education and tasting	1	6·3	11–20	4	25·0
In development	2	12·5				Education, tasting, pupils take home	6	37·5	21–30	4	25·0
Not stated	1	6·3				Education, pupils take home	1	6·3	31–40	2	12·5
						Education, tasting, pupils take home, canteen	3	18·8	Not stated	1	6·3
						Don’t have one/ not used	3	18·8			
Does policy include break snack advice?			Food tasting sessions held at school?			Food given to parents at school events?	13	81·3	Types of food commonly wasted?		
Yes guidance	7	43·8	Yes, regularly	2	12·5	T/C +/or sweet foods (biscuits, buns, dessert)	6	46·2	Potatoes	9	56·3
Yes requirements	6	37·5	Yes, ad-hoc	10	62·5	T/C/juice +/or sweet foods, fruit	3	23·1	Vegetables	8	50·0
In development	2	12·5	No	4	25·0	T/C +/or sweet foods, sandwiches, hot meal	2	15·4	Bread, rice, pasta	5	31·3
Not stated	1	6·3	Types of foods at food tasting sessions?			BBQ items	1	7·7			
			Special occasions	4	33·3	Sample of school meals	1	7·7			
			Food from other cultures	3	25·0						
			Linked with curricular topics	6	50·0						
			School programmes (FAST/ STEM)	3	25·0						
			Health and fitness week	1	8·3						
How is policy enforced?			Fruit and vegetable sampling	1	8·3	Parental involvement in food activities?			Do pupils give feedback on meals to caterers?		
Staff encourage pupils to keep policy	7	43·8				School events	5	31·3	Yes (e.g. pupil councils)	7	43·8
Parents reminded of policy if breached, pupils cannot eat unsuitable foods	4	25·0				Bring an adult to lunch days	1	6·3	No	8	50·0
Not stated/ not clear	5	31·3				Parents bring in food from their cultures	1	6·3	Not stated	1	6·3
						No/ not clear/ not stated	9	56·3			

Some schools monitored or encouraged food intake during lunchtime (*n* 8, 44 %) (Table 1). In these eight schools, caterers encouraged pupils having school meals to take full meals and try fruit and vegetables (*n* 4, 50 %), verbally praising this (*n* 1, 13 %). Canteen supervisors monitored and encouraged pupils to eat their school meal or packed lunch (*n* 4, 50 %). At one school, designated senior pupils awarded stickers to other pupils in the canteen at lunchtime that brought ‘healthy’ packed lunches that complied with school healthy eating policy to school with them that day (*n* 1, 13 %). Several schools (*n* 13, 73 %) promoted nutrition messages through poster displays.

### Food provision

Menu options influenced pupils’ decision to have a school meal, with chips being the most popular menu item (*n* 12, 75 %), followed by chicken curry and roast dinners (both *n* 6, 38 %) (Table 2). School meal food waste was estimated at 5–40 %, with > 50 % of schools stating potatoes (*n* 9, 56 %) and vegetables (*n* 8, 50 %) were commonly wasted. At just under half of schools (*n* 7, 44 %), pupils offered feedback on school meals to caterers, e.g. through pupil councils.

Many schools had a breakfast club (*n* 14, 88 %), providing foods through school funding or for purchase. All served cereal and toast or breads, with variation in additional foods offered. Half of the schools (*n* 8, 50 %) provided healthy break-time snacks for pupils through school funding or for purchase. At one school, pupils could purchase fruit after school (*n* 1, 6 %). More than half of schools ran *ad hoc* tasting sessions (*n* 10, 63 %) and cookery clubs (*n* 9, 56 %). Most schools held themed food days (*n* 14, 88 %) based on special events, e.g. Pancake Tuesday or fun days (*n* 5, 36 %), trying food from other cultures (*n* 3, 21 %), linked with curricular topics (*n* 1, 7 %) or a combination (*n* 4, 29 %). Most schools (*n* 13, 87 %) used school allotments for education, tasting and growing produce to take home. Food most commonly provided at school events was tea/coffee and biscuits, buns and desserts (*n* 6, 46 %).

### Parental engagement in school food

In addition to communicating food policies, some schools also engaged parents in food activities, including school events (*n* 5, 31 %), invitation to a school meal with pupils (*n* 1, 6 %) and parents bringing food from their culture into school for pupil tasting (*n* 1, 6 %) (Table 2).

### Whole-school food environment recommendations based on current strengths and areas for enhancement

Areas of good practice and where there was scope for change were apparent (Tables 1–2). Key areas of strength in implementing a WSFE were defined as areas implemented by > 50 % of schools and included measures in place to enhance physical eating environments, healthy eating policies with reinforcement via a range of meals and a range of school food activities such as tasting sessions and cookery activities. Areas of a WSFE approach that that could be enhanced were also identified (defined as implemented by ≤ 50 % of schools). These were combined and summarised as recommendations in Table 3.

**Table 3:** Recommendations based on primary school best practice for achieving an optimal primary school food environment

Step		Examples of how to implement step
1	Provide an inviting dining area with a relaxing atmosphere	Simple, low-cost changes, e.g. use of bunting, tablecloths and centre pieces can create a welcoming and comfortable café style environment. Introducing systems to reduce busyness (e.g. multiple serving points), allow time to eat (e.g. review length of lunch break or allocate specific eating and play times) and allow pupils to socialise with their friends while eating (e.g. packed lunch and school meal pupils siting together)
2	Present food attractively and offer food tasting	Present food in creative ways through use of serving platters, stands, individual serving portions, creative food cutting equipment or a salad trolley, display the daily menu and label or display foods on offer and allow pupils to taste foods
3	Reinforce healthy eating messages in the dining area	As well as displaying posters on healthy eating, catering staff could encourage eating such as taking all components of the meal or trying vegetables. Additionally, adults could eat in the canteen with pupils as role models and to promote conversation about food (e.g. rota for pupils parents or teaching staff)
4	Monitor adherence to break and packed lunch policies	Staff checking food brought in, contacting pupils parents if breached
5	Reinforce healthy eating through school food activities	Reinforce healthy eating through school food activities such as cookery club, chef demonstrations, food tasting sessions, themed food days in canteen and in the classroom and consider introducing school food activities with a healthy eating focus if not existing
6	Provide healthy food options at school events	Offer healthy food options at school events such as school plays, sports days, BBQ
7	Encourage parental involvement in school food	Inviting parents to school lunch, school food activities such as cookery and school food events such as themed food days
8	Promote pupil ownership of school food	Involve pupils in the process of planning school meals and allow them to give feedback on meals to catering staff as customers

### Use of instrument to capture whole-school food environments

Both the observation proforma and questionnaire captured intended detail for most items. Some questionnaire items, however, were less well completed including items that required quantification and open-ended items on food policy enforcement and parental involvement. Additionally, the open-ended item to capture detail on themed food days largely did not capture specific foods offered at these events. It was apparent through analysing the questionnaire data that questions could be added to a number of areas covered including on FSM and food waste to capture further detail.

## Discussion

To date, SFE research has largely focused on the presence of dining facilities and food provided within the canteen setting^(14)^. It is apparent from findings of a recent systematic review of methods of measuring the SFE that many existing instruments are designed to measure specific elements of the SFE, such as these physical attributes^(8)^. WSFE approaches to healthy eating are recommended^(15)^. The developed instrument therefore builds on existing tools to capture detail on WSFE, including the physical setting, food policies, all food provision and parental engagement. The developed instrument is useful from a research perspective for capturing WSFE in primary schools, informing future interventions to improve children’s dietary behaviour and capturing effectiveness of interventions designed to improve SFE in research settings. To illustrate this, we captured data at baseline from schools involved in a food-based intervention^(12)^ and determined areas of strength or where improvement was required (using an arbitrary cut-off of 50 % of schools to determine this). The developed instrument could also formally be adapted for self-assessment use by schools to provide evidence of meeting whole-school food guidance, thereby encouraging best practice and consistency in food environments across schools.

Findings highlight that WSFE vary between participating schools, with differences in physical settings, food policies, food provision and level of parental engagement. Most schools had healthy eating policies, in line with existing literature^(16)^, but there was variation in policies and scope for enhanced policy implementation and monitoring. The need for consistent school food policies and monitoring of policy implementation has been acknowledged^(17)^. Food provision in the canteen and policies for foods brought into school aligned with school food standards. It was observed that healthy dietary behaviour could be further promoted through other areas of school food provision such as cookery activities and food-related events. There was also scope to enhance the physical setting (including food presentation) and parental involvement in school food. Consistent with these findings, school-based interventions to improve children’s dietary behaviour often incorporate cookery activities, food tasting, food-related events and elements to enhance food presentation^(18,19)^, suggesting that these are areas of the WSFE that could be further enhanced. Additionally, parental involvement has been identified as an element of whole school approaches to healthy eating that needs to be strengthened^(20)^. Success of whole-school approaches for encouraging healthy dietary behaviour have been demonstrated^(15)^, which highlights the value of schools making small changes to enhance the key food environment elements, and areas of good practice in implementing a WSFE, which were apparent at all schools, should be shared across schools.

The main limitation of the instrument is that it did not undergo validity and reliability testing beyond initial face validation by the research team. Additionally, suggested modifications to the instrument were outlined after data collection and analysis were complete. For example, the observation proforma largely included open-ended items to capture detail. Responses obtained for these items could be used to develop closed-response items to aid ease of future completion and analysis. A subjective indication of perceived acceptability of canteen queue times was captured. Quantifying queue times would provide further information, however, this would require additional research personnel during the observation and may be complex to capture. Perception of general food waste was captured through the questionnaire; however, an objective measure of food waste could be incorporated into the observation proforma to provide more detail on this issue and could capture kitchen and plate waste. Objectively measuring food waste would also require additional research personnel. Areas where further questions could be added to the questionnaire were highlighted. Specific food offered at themed food days could be assessed using an additional item. The free school meal (FSM) question asked whether FSM-eligible pupils took FSM, but a question could be added regarding what % of pupils were eligible for FSM which would provide context. Questions could also be added to capture participation in local community-focused food-based activity and school policy on celebratory/reward foods. While we aimed to capture many elements of the whole-school environment, it was not designed to measure food-related education or surroundings of the school, which would be important elements to add for future work.

Next steps to improve the instrument for future use would necessitate incorporating these suggested edits. To enhance the robustness of the instrument for use as a research tool, validity and reliability assessments would need to be conducted following amendment of the instrument, such as establishing face validity through review of the instrument by an expert panel external to the research team and conducting psychometric assessments such as test-re-test reliability of the instrument. The following next steps could also be implemented for future use of the instrument by schools for self-assessment of meeting school food guidance. A scoring protocol could be developed, perhaps also linked to identification of barriers and facilitators to change and, ultimately, lead to tailored recommendations for schools, always including the requirement for consultation with stakeholders before implementation. As an example, previous studies identified fiscal constraints as a significant barrier to implementation of such policies, whereas the provision of financial support was identified as a facilitator^(16)^. The instrument could be adapted for a similar range of applications in secondary schools. The developed instrument could be extended to include consideration of cost of changes to policy. Such an instrument would be more comprehensive and detailed than the checklist (with tick-boxes) aimed at principals to aid the adoption of a whole-school food approach that is already available for schools as guidance for inspection preparation^(21)^. These next steps could ultimately enhance the sustainability of healthy SFE and optimise impact of such policies on pupils’ dietary behaviours.

## Conclusion

A research instrument was developed and tested to measure WSFE in the primary school setting. The instrument captured desired detail, although potential modifications were identified and further validity and reliability testing of the instrument is required for future use as a research tool. Future adaptations of the instrument were also identified, such as for self-assessment use by schools. Using the instrument to measure current food environment across a range of schools enabled identification of recommendations that could be implemented to enhance SFE.

## Supplementary material

For supplementary material accompanying this paper visit https://doi.org/10.1017/S1368980023001131. click here to view supplementary material
