# Supplementary material for: Capturing the whole-school food environment in primary schools
Source: Public Health Nutr. 2023 Jun 5;26(8):1671–8. doi: 10.1017/S1368980023001131 (PMC10410367; doi:10.1017/S1368980023001131)
Supplement: Supplementary file 1 [file S1368980023001131sup001.docx]

**Supplementary material – Whole-school food environment measurement tool**

1. **Observation proforma** (completed by researcher)

| **Canteen environment** | |
| --- | --- |
| Is the canteen area welcoming? (Clean/ attractive colours/ comfortable seating/ adequate space and seating)? |  |
| Atmosphere (Busyness/ noise/ silence/ music/ temperature/ smell/ lighting)? |  |
| Set meal served/ choice? Snack bar available? |  |
| Meal served to pupils or pupils queue? |  |
| Reasonable queue times? |  |
| Singular/ multiple serving points? |  |
| Menu/ options displayed?  Foods labelled? |  |
| Is food available displayed? |  |
| Posters/ displays about food? |  |
| What is food served on? Plate/ segmented tray? |  |
| Is food presented in a way that is appealing to children? (e.g. use of serving cups/ chopped up) |  |
| Is seating arranged or can pupils sit anywhere? |  |
| Do pupils eating packed lunches and school meals sit together? |  |
| Do teachers eat school meals? Do they eat in the canteen with pupils? Are there other adults present, e.g. classroom assistants? How do these adults interact with the pupils during lunch/monitor/encourage their intake? |  |
| Dining hall supervisors? Role? |  |
| Length of lunch break? Sense of rush in eating? |  |

1. **Questionnaire** (Completed by School Senior Management with catering staff input)

| **SCHOOL FOOD POLICY** | |
| --- | --- |
| Q1 | Does your school have a specific healthy eating policy?  **□** Yes, written policy in place **(Go to Q2)**  **□** Currently developing a written policy **(Go to Q8a)**  **□** No **(Go to Q8a)** |
| Q2 | Please describe briefly how the policy was developed (e.g. Food in Schools Policy or school specific policy) |
| Q3 | How long has the policy been in place? **________** years |
| Q4 | Which of the following methods are used to communicate your school’s healthy eating policy to parents? **(Please tick all that apply)**  **□** Paper or electronic copy distributed  **□** Verbal communication (e.g. parents evening)  **□** Publication online via school website  **□** Publication online via social media (e.g. Facebook)  **□** Posters/signs around school  **□** School newsletter  **□** Other (Please state): **_____________________________________** |
| Q5 | Does your healthy eating policy include guidance or requirements on the types of foods and beverages that pupils can bring into school in packed lunches? **(Please tick one box only)**  **□** Yes, guidance  **□** Yes, requirements  **□** No  **□** Don’t know |
| **SCHOOL FOOD POLICY (Continued)** | |
| Q6 | Does your healthy eating policy include guidance or requirements on the types of foods and beverages that pupils can bring into school for break time? **(Please tick one box only)**  **□** Yes, guidance  **□** Yes, requirements  **□** No  **□** Don’t know |
| Q7 | If your healthy eating policy includes guidance or requirements on the types of foods and beverages that pupils can bring into school at break or lunch, is it enforced? If yes, how is it enforced? (Please state) |
| **SCHOOL FOOD ACTIVITIES** | |
| Q8a | Does your school offer a breakfast club?  **□** Yes **(Go to Q8b)**  **□** No **(Go to Q9a)** |
| Q8b | What types of foods are available to pupils at breakfast? (Please state) |
| Q8c | How many pupils take breakfast each day on average? **___%** of total pupils |
| Q9a | Is food provided for pupils at break time?  **□** Yes **(Go to Q9b)**  **□** No **(Go to Q10a)** |
| **SCHOOL FOOD ACTIVITIES (Continued)** | |
| Q9b | What types of foods are available to pupils at break time? (Please state) |
| Q10a | Is food provided for pupils after school?  **□** Yes **(Go to Q10b)**  **□** No **(Go to Q11a)** |
| Q10b | What types of foods are available to pupils after school? (Please state) |
| Q11a | Does your school offer a supper club?  **□** Yes **(Go to Q11b)**  **□** No **(Go to Q12a)** |
| Q11b | What types of foods are available at the supper club? (Please state) |
| Q12a | Does your school hold food tasting sessions for pupils?  **□** Yes, regularly **(Go to Q12b)**  **□** Yes, ad-hoc/ one off **(Go to Q12b)**  **□** No **(Go to Q13a)** |
| Q12b | What types of foods are available for pupils to try at food tasting sessions? (Please state) |
| **SCHOOL FOOD ACTIVITIES (Continued)** | |
| Q13a | Does your school hold themed food days or events? (e.g. International food day)  **□** Yes, regularly **(Go to 13b)**  **□** Yes, ad-hoc/ one off **(Go to 13b)**  **□** No **(Go to 14a)** |
| Q13b | What type of themed food days/events are held at your school and what types of foods are offered at these? (Please state) |
| Q14a | Does your school have facilities/ equipment to hold a cookery club or demonstration for pupils?  **□** Yes **(Go to Q14b)**  **□** No (**Go to Q15a)** |
| Q14b | Does your school hold a cookery club for pupils?  **□** Yes, regularly **(Go to Q14c)**  **□** Yes, ad-hoc/one off **(Go to Q14c)**  **□** No **(Go to Q15a)** |
| Q14c | What do pupils make at the cookery club? (Please state) |
| Q15a | Does your school hold chef/cookery demonstrations for pupils?  **□** Yes, regularly **(Go to Q15b)**  **□** Yes, ad-hoc/ one off **(Go to Q15b)**  **□** No **(Go to Q16a)** |
| **SCHOOL FOOD ACTIVITIES (Continued)** | |
| Q15b | What type of meals/ recipes are demonstrated? (Please state) |
| Q16a | Does your school have gardens/ an allotment?  **□** Yes **(Go to Q16b)**  **□** No (**Go to Q17a)** |
| Q16b | How are foods grown in the school garden/ allotment used? **(Please tick all that apply)**  **□** Education  **□** Taste testing  **□** Pupils take home  **□** Used in school canteen  **□** Other (Please state) **____________________________________** |
| Q17a | Is food offered to parents and pupils at school events?  **□** Yes **(Go to Q17b)**  **□** No (**Go to Q18)** |
| Q17b | What type of food is offered at school events? (Please state) |
| Q18 | Are parents involved in any school food activities? If yes, please state which activities: |
| **SCHOOL FOOD ACTIVITIES (Continued)** | |
| Q19 | Are there any other food related activities currently existing in your school? If yes, please state the activities: |
| Q20 | Are there any other food related activities that you would like to start at your school? If yes, please state the activities: |
| **SCHOOL MEALS** | |
| Q21 | How many pupils take school meals per day on average? **__%** of total pupils |
| Q22 | How many free school meal entitled pupils take school meals? **____**% of entitled pupils |
| Q23 | Are there particular days when more pupils take a school meal due to the menu choice?  **□** Yes  **□** No |
| Q24 | What meals are most popular on the menu? (Please state) |
| Q25 | On a scale of 0-100%, how much food waste do you think there is in your school canteen? **______**% |
| Q26 | What types of food (if any) are most commonly wasted in your school canteen? (Please state) |
| Q27 | Do pupils provide feedback on school meals to catering staff? (e.g. via a pupil council)  **□** Yes  **□** No |
| **Thank you for completing this questionnaire** | |
